# Case of Paradoxical Cultural Sensitivity: Mixed Method Study of Web-Based Health Informational Materials About the Human Papillomavirus Vaccine in Israel

**DOI:** 10.2196/13373

**Published:** 2019-05-17

**Authors:** Nour Abed Elhadi Shahbari, Anat Gesser-Edelsburg, Gustavo S Mesch

**Affiliations:** 1 School of Public Health University of Haifa Haifa Israel; 2 Department of Sociology University of Haifa Haifa Israel

**Keywords:** web-based health informational materials, HPV vaccine, Israel, cultural sensitivity, transparency, sexuality, quantitative analysis, qualitative content analysis, Hebrew and Arabic

## Abstract

**Background:**

Designing web-based informational materials regarding the human papillomavirus (HPV) vaccine has become a challenge for designers and decision makers in the health authorities because of the scientific and public controversy regarding the vaccine’s safety and effectiveness and the sexual and moral concerns related to its use.

**Objective:**

The study aimed to investigate how cultural sensitivity (CS) is articulated in the explanatory informational materials on the HPV vaccine that are posted on the websites of the Israeli health authorities. In addition, the study examined the effect of transparency on the expression of CS in the informational materials.

**Methods:**

The study employed a quantitative and qualitative content analysis of the texts of explanatory informational materials published on the Arabic and Hebrew websites of the Israel Ministry of Health and the Clalit health maintenance organization (HMO).

**Results:**

The findings revealed the differences in the dimensions of CS (based on the CS model by Resnicow) between the informational materials targeting the majority Jewish population and those targeting the minority Arab population. Indeed, the research findings point to a paradox. On the one hand, the materials appealing to the conservative Arab population exhibited CS, in that the sexual context of the vaccine was missing. On the other hand, analysis of Resnicow's deep dimensions showed that disregarding the sexual context does not allow the relevant target audience to reflect on the barriers and concerns. In addition, the way the information was provided exhibited a lack of transparency regarding the CS dimensions (surface and deep).

**Conclusions:**

The public health authorities have 2 main objectives in the context of vaccinations. One is to raise the vaccination rates and the other is to provide full and culturally sensitive information to give the public the tools to make intelligent decisions. The findings of this study indicated that despite the high uptake rate for HPV vaccination in the Arab population, the health authorities did not exercise full transparency and CS in transmitting the association between engaging in sexual relations and the necessity of the vaccination. Thus, the major challenge for the health authorities is to find ways to implement the objective of communicating information about the vaccination in a way that is transparent and culturally sensitive, even if this raises questions and fears among the public deriving from their culture.

## Introduction

### Health Promotion and Cultural Sensitivity

Individual and cultural differences (attitudes, intentions, beliefs, subjective norms, and the like) moderate the effects of health promotion interventions and influence how individuals make decisions [1].

Segmentation and determination of a specific target audience aim to identify the characteristics, needs, social structures, and perceived costs and benefits of the audience relative to specific health behavior. Identifying all these factors in the target audience can contribute to the development of an effective health communication [2].

Studies show that messages are more thoroughly processed and defined as convincing and effective if their content is adapted to the cognitive, emotional, and motivational characteristics that in most cases are determined by the cultural background of the target audience [3].

Culturally sensitive materials based on the target population’s cultural background can affect the understanding and effectiveness of health communication [4]. Moreover, culturally sensitive materials increase the awareness of recommended behavior, improve decision making, make messages more persuasive, and lead to deeper processing and understanding in the context of health promotion. Sensitive cultural communication reduces gaps in health outcomes by making messages equally meaningful and effective [5].

When the information provided matches cultural values, the content is likely to lead to deeper reflection on the potential benefits and adverse effects, in turn leading to more appropriate and informed choices in the decision-making process [6].

Despite the emphasis on the importance of audience segmentation and theory-driven development of messages (cultural sensitivity [CS] in this study), health organizations do not always take the dimensions of CS into consideration when designing interventions and formulating messages and informational materials [7,8].

### Resnicow’s Model of Cultural Sensitivity

Culture is a typical mediator in the decision-making process of individuals with respect to the recommended health behavior [9-11]. CS is defined as the extent to which the ethnic, cultural, and experiential norms, values, behavior patterns, and beliefs of the target population, as well as the historical, environmental, and social factors are expressed in the design, planning, and evaluation of the informational materials and programs for health promotion [12].

Many theories and models have discussed cultural competence and CS to various ethnic minority groups in different intervention programs [7,13-16]. The cultural competence or CS of materials used for health education and publicity is measured by different tools focusing on the use of culturally adapted language, graphics, and illustrations as well as the use of personages from the community [9,17].

Over the years, many studies have shown that CS is not limited to language or illustrations, suggesting that deep dimensions require additional measures. According to Resnicow's definition [15], CS is conceptualized in terms of 2 main measures: *deep dimensions* and *surface dimensions*.

*Surface dimensions* include the use of people, places, language, music, food, and clothing familiar to the target audience. The measure also includes channel identification (media), level of literacy, relevance of recommended behavior to the target audience, marketing sites (eg, schools and churches) appropriate for transmitting messages and intervention programs, and the use of a suitable communicator [12].

These surface dimensions are intended to make communication more effective by increasing its attractiveness, relevance, and familiarity as well as by increasing source reliability with respect to the target population [1].

*Deep dimensions*, in contrast, examine the environmental, cultural, social, psychological, and historical factors related to the target audience that influence their health behavior. The measure makes specific reference to the arts, values, shared experiences, moral concerns, cultural barriers, perceptions regarding those who determine behavior, and perceptions regarding recommended behavior [15].

These dimensions are related to the understanding and interpretation of health communication, which depend on the models that explain the health and illness status of the target population [15].

### Cervical Cancer and the Human Papillomavirus Vaccine

The human papillomavirus (HPV) is one of the most common sexually transmitted infections worldwide [18,19]; this contrasts with the situation in Israel, where morbidity and mortality rates caused by HPV infections are the lowest in the West [20]. Some studies indicate that ongoing infection with high-risk HPV strains can develop into cervical cancer. HPV can also cause other cancers (cancers of the vagina, vulva, penis, anus, rectum, and oropharynx) and genital warts [21-23]. High-risk HPV strains include HPV 16 and 18, which cause approximately 70% of cervical cancers [22,24].

In addition to the Papanicolaou (Pap) smear screening procedure for cervical cancer that has significantly reduced the morbidity and mortality rates for cervical cancer in many western countries [25], the HPV vaccine is the first vaccine targeted at preventing cervical cancer [26].

According to the World Health Organization (WHO), since 2006, around 100 nations worldwide have approved the HPV vaccine, and as of 2012, approximately 40 countries have included the vaccine in their immunization programs [27]. HPV vaccine programs vary from country to country with respect to the type of vaccine, implementation strategies, compliance rates, and payment policies [28-30].

According to recent data from the Israel Ministry of Health [20], the age-adjusted incidence rates (per 100,000) of invasive cervical cancer in 2012 were 5.08 in Jewish women and 2.97 in Arab women. The age-adjusted incidence rates (per 100,000) of stage 0 cervical cancer in 2012 were 22.5 in Jewish women and 4.2 in Arab women. The age-adjusted mortality rates (per 100,000) from cervical cancer in 2012 were 1.50 in Jewish women and 0.94 in Arab women. The data show that the rates of HPV infections are even lower.

Starting in 2007, the HPV vaccine was available in Israel in exchange for payment. In December 2012, Israeli health authorities decided to include the HPV vaccine in the medical services basket of medical services provided free of charge. The vaccine included in the basket is for women, boys, and men up to the age of 26 years, who constitute the population at risk for HPV infection [31]. Starting in 2013, the vaccine was included as a part of the routine vaccines given in school to girls in the eighth grade and was later extended to include boys [31,32]. According to the Ministry of Health [20], in the 2016 school vaccination campaign, the rate of HPV vaccine uptake among the Arab population reached 84% (96% in the Bedouin sector) compared with 40% for the Jewish population.

### Transparency Regarding the Human Papillomavirus Vaccine

Experts from across the globe recommend additional monitoring of HPV vaccine effectiveness and safety [33]. This recommendation derives from the scientific controversy regarding the effectiveness and safety of this vaccine since its inclusion in vaccination programs worldwide. On the one hand, a large body of studies indicate that the vaccine is effective. For example, various meta-analyses and research studies have attested to the vaccine's high immunological effectiveness, approaching 93% to 100%, against the papilloma virus and other precancerous cervical lesions [28,34-38]. In addition, recent research emphasizes the effectiveness of the HPV vaccine in reducing preinvasive cervical disease and increasing clinical herd protection in unvaccinated women [39]. Yet, the literature still includes studies pointing to a variety of factors that can affect the vaccine’s effectiveness, such as age, the type of virus strain, and the individual’s immune system [40-44].

On the other hand, other studies point to negative consequences after vaccine uptake. Similar to any other vaccine, the HPV vaccine can cause major pain at the injection site as well as fainting and dizziness [45-47]. However, there have also been reports of more serious side effects [48-59]. With the rising number of these reports, experts around the world have been calling for more studies and improved postmarketing surveillance [56,59].

Balanced health information regarding the HPV vaccine should include 3 main components: (1) completeness—description of the risk of cervical cancer and the vaccine’s advantages and disadvantages; (2) transparency—presentation of all the vaccine-related risks in absolute numbers and not as relative figures; and (3) evidence-based correctness—providing information based on science [60].

Transparency is an essential component in effective health communication and in designing messages and explanatory materials intended to promote various health behaviors [61-64]. Studies that examined transparency in providing information on the HPV vaccine to target populations were conducted mainly in Germany [60,65]. These studies emphasized that the German health agencies and the German media as well provided only partial information regarding the risks of cervical cancer and the papilloma virus and used statistics that were not transparent in describing the vaccine’s effectiveness and safety. The statistics in the marketing materials provided inaccurate numbers regarding the vaccine’s effectiveness, showing absolute effectiveness. Moreover, most of the marketing leaflets did not mention the vaccine’s side effects [60,65]. Most studies examining decision making in the context of the HPV vaccine emphasize the importance of transmitting information that is fully transparent and understandable to enable parents to sign the informed consent forms for the vaccination [66-68]. Some studies indicate that people do not always read the forms to the end or do not understand what they read, so, in essence, they sign the forms without their informed consent [66,69,70]. Moreover, most of the scientific literature indicates that in the case of the HPV vaccine, the parents indicate that their knowledge about the vaccine is limited [71-74]. This makes the informed-consent decision-making process to the HPV vaccine more challenging [71,75-77]. The studies show that people often make decisions about HPV vaccination without having sufficient knowledge as required to make age-appropriate health decisions [74,77-82].

Furthermore, providing complete, comprehensive, and transparent information about HPV vaccination helps parents in making a decision about whether to vaccinate their children [66,67]. Parental decision making regarding the vaccination is dependent upon perceptions of the risks with respect to their children’s sexual activity [72,74,82,83]. Studies indicate that parents who perceived the risk of sexual activity among their children to be high were more willing to give their children the vaccination. In contrast, parents who perceived the risk of sexual activity among their children to be low because of their cultural or religious background were less willing to give their children the vaccine [72,73,75,79].

### Human Papillomavirus Vaccine and Sexuality

The HPV vaccine is intended to protect the individual from contracting the papilloma virus, which is transmitted through sexual intercourse [19]. The *Food and Drug Administration* recommends vaccinating boys and girls against the virus during adolescence, before they become sexually active [84]. Research studies have raised parental concerns and fears regarding the vaccine from different population groups worldwide because of their belief that the vaccine legitimizes sexual activity and promotes sexual abuse [85-87]. Some of these concerns were derived from media coverage that represented giving the vaccine as an act that encourages sexual relations, sexual promiscuity, and sexual abuse [88,89]. Despite these parental concerns, several scientific research studies showed that the HPV vaccine has no impact on the sexual behavior of adolescents [90-93]. Nonetheless, the issue of sexual relations in the context of giving the vaccine increases sensitivity regarding the vaccine [94], particularly among conservative minority population groups such as the Arab minority in Israel [95,96].

### Arab Population in Israel and the Context of Sexuality

Constituting around 20% of the general population, the Arab population in Israel is a minority with unique national, religious, linguistic, and cultural attributes. Relations between Arabs and Jews are mainly minority-majority relations. Israel must deal with the internal problems of minority groups for whom issues of justice and equality are central to their relations within the state [97].

The socioeconomic disparities between the Jews and the Arabs are the result of a historical-geographic reality in which most of the Arab population lives in the spatial periphery, thus limiting their access to the country's main labor markets and services. These disparities find expression in unequal allocation of resources for economic development and infrastructure in the local authorities and unequal employment opportunities for Arabs [97].

In addition, the culture of Arab society differs from that of Jewish society. These cultural differences are related to language, religion, nationality, cultural heritage, family structure, values, and primary lifestyles. Traditionalism dominates Arab society, in that the religion is a central component of cultural life (religious self-definition and prayer frequency). Moreover, the Arab population still lives within the framework of a conservative family and community and is characterized by less openness, autonomy, development of personal inclinations, freedom, and the like. This is not the case among the predominantly secular Jewish population, which espouses individualism, competitiveness, achievements, ambitions, career development, materialism, and consumerism [97].

Even though the Arabs in Israel usually live in separate localities marked by lower educational and socioeconomic levels, their exposure to Jewish society is high, which has led to social, cultural, and economic processes of change that have made Arab society more modern. This modernization is characterized by a gradual shift from a collectivist to an individualist orientation, manifested in a rise in the age at first marriage, a drop in the number of children, an increase in the educational level, use of preventive health services, and more years of education among women than among men [98].

Yet, Arab culture is still conservative regarding sexuality and sees sexual relations before marriage as improper and prohibited. Indeed, in most Arab communities, such relations are taboo [99-101]. One of the typical barriers preventing parents from the Arab minority in Western countries from vaccinating their daughters against the papilloma virus is their concern of encouraging unlimited sexual behavior and sexual promiscuity [102-104].

To the best of our knowledge, no studies have examined the topic of CS in explanatory materials geared at promoting the administration of HPV vaccine. Therefore, the purpose of this study was to investigate the following question: How is CS articulated in the explanatory informational materials about the HPV vaccine posted on the websites of Israeli health authorities?

To investigate how CS is articulated in the explanatory informational materials about the HPV vaccine posted on the websites of the Israeli health authorities, we examined 3 specific questions:

1. How are the surface and deep dimensions of CS from Resnicow’s model articulated in the explanatory informational materials posted in both languages (Hebrew and Arabic)?
2. Are the surface and deep dimensions of CS articulated differently in materials intended for the Jewish population than in those intended for the Arab population?
3. Is there a difference between the materials written in Arabic and those written in Hebrew with respect to transparency in transmitting complete information?

## Methods

### Study Sample

The research method entailed quantitative and qualitative content analysis of the texts of explanatory informational materials published on the Arabic and Hebrew websites of the Israel Ministry of Health and the Clalit HMO.

The research was conducted under the approval of the Faculty of Social Welfare and Health Sciences Ethics Committee for research with human subjects at the University of Haifa (approval no.118/16). The researchers analyzed these materials for 6 months (October 2017-March 2018).

The sample comprised 18 instances of explanatory materials published on the Hebrew and Arabic websites of the Clalit HMO and the Ministry of Health and updated after the vaccine was included in the basket of health services (after 2012). These instances had identical content and headings in Hebrew and in Arabic (9 in each language).

Excluded from the study were materials published on sites that did not have an Arabic website, websites that had not been updated after the vaccine was included in the Israeli basket of health services (2012) and those that had original materials from the WHO that were not translated into Hebrew or Arabic and were omitted because of the Arab population’s low level of literacy in English [105].

The rationale behind selecting all the explanatory informational materials for the HPV vaccine after it was included in the medical services basket is as follows. Although the vaccine is free and available in school settings, parents are still aware of the scientific controversy surrounding the vaccine’s efficacy and effectiveness as publicized in the media. Therefore, they still seek information about the vaccine, its efficacy, and its safety in deciding whether to vaccinate their children [33].

The following health authority websites and the HPV vaccine materials were retrieved:

1. Ministry of Health: Human Papilloma Virus Vaccine, Information Sheet Before and After Administering HPV Vaccine, HPV Vaccine for Eighth Grade Girls, HPV Vaccine for Eighth Grade Boys, Vaccine to Protect Against Cervical Cancer Caused by Papilloma Virus, PowerPoint Presentation about HPV Vaccine.
2. Clalit HMO: Cervical Cancer (first document), Cervical Cancer (second document).

The following keywords were examined: cervical cancer, vaccine against cervical cancer, vaccine against the human papilloma virus, human papilloma virus, condyloma, and genital warts.

It is important to clarify that these sites address most of the Jews and Arabs living in Israel. Most of the Jewish population is secular and, therefore, tends toward more liberal views, except for the conservative ultraorthodox Jews who are in the minority [106]. Similarly, the majority of the Arabs in Israel are Muslim (97% of the Arabs in Israel), who are indeed defined as conservative [107].

### Analysis

The explanatory materials were analyzed using 2 parallel methods: quantitative content analysis based on index-coding criteria and qualitative content analysis to interpret and explain the index score.

#### Cultural Sensitivity Index Coding

Quantitative content analysis is based on coding. According to this method, the analysis begins by identifying relevant concepts based on a theory, model, or previous studies that form the basis of the index-coding scheme. The next step is to determine the index-coding scheme by developing classification rules to assign coding units to specific categories or concepts. The coding guide ensures systematic encoding and allows data recovery. In addition to the coding guide, a coding form is also used to record the details of the codes that apply to the data during the coding process [108].

##### Building the Cultural Sensitivity Index

On the basis of Resnicow's theoretical definitions of 2 dimensions, deep dimensions and surface dimensions [15], we constructed an index of appropriate categories that served in analyzing the CS of the explanatory materials intended for the Jewish and Arab populations in Israel.

The index was constructed using 2 dimensions: deep and surface dimensions. For each of these dimensions, we designed nominal categorical indicators that can take 1 of 2 values: yes/no (see Textboxes 1 and 2). If the explanatory materials do not provide full and transparent information with respect to a specific indicator—whether for surface dimensions or for deep dimensions—the indicator is scored as *no*. Conversely, if the explanatory materials provide full and transparent information, the indicator is scored as *yes*.

**Surface dimension index: Used to analyze the explanatory health informational materials of the human papillomavirus vaccine intended for the Arab and Jewish population in Israel with nominal categorical indicators (yes/no).**

**Surface dimension categories:**

- Is the target population mentioned in the title or the content, either directly or indirectly?
- Do the materials use people who are appropriate for, familiar to, and acceptable to the target population?
- Do the materials use the mother tongue of the target population?
- Are the materials formulated in a manner appropriate to the literacy level of the target population?
- Do the materials explain the relevance of cervical cancer to the target population?
- Do the materials explain the effectiveness of the human papillomavirus (HPV) vaccine against cervical cancer in the target population (safety, immunity, etc)?
- Do the materials explain the implications of the HPV vaccine against cervical cancer in the target population (side effects, complications, etc)?
- Are appropriate media channels used that are familiar to the target population?
- Are the materials distributed in places most suitable for the target population (churches, schools, medical clinics versus websites)?
- Do the materials use a reliable communicator who is appropriate for the target population?
- Was the organization that transmitted the information from the same ethnic group (Arab/Jewish population)?

**Deep dimension index: Used to analyze the explanatory health informational materials of the human papillomavirus vaccine intended for the Arab and Jewish population in Israel with nominal categorical indicators (yes/no).**

**Deep dimension categories:**

- Do the materials make reference to the moral concerns of the target population regarding the human papillomavirus (HPV) vaccine (increased sexual relations, encouraging promiscuity)?
- Do the materials make reference to the cultural concepts regarding cervical cancer and the HPV vaccine (preference for women practitioners for Pap smears or other gynecological exams)?
- Do the materials make reference to social concepts regarding cervical cancer and the HPV vaccine (cervical cancer is a female disease, reasons for giving the vaccine to males)?
- Do the materials refer to environmental concepts (influence of the Jewish/Arab population, socioeconomic status)?
- Do the materials make reference to value concepts (religion, fatalism, and sexual relations before marriage)?

#### Cultural Sensitivity Index Scores

The specific dimension score (%) was calculated by the ratio between the number of *yes* answers on each specific measure and the total indicators for each dimension. For example, to calculate the score for the surface dimension, the number of *yes* answers is divided by 11 (the total number of surface dimension indicators).

To calculate the score for the deep dimension, the number of *yes* answers is divided by 5 (the total number of deep dimension indicators).

The overall CS index score (%) is calculated by the average sum of the surface dimension and the deep dimension score (CS=1/2 × surface dimension score + deep dimension score).

The index yields 2 scores: a specific index score and a general score. These 2 scores facilitate direct comparison of the explanatory materials in terms of CS scores and diagnosis of level of cultural appropriateness of materials targeting the Arab and Jewish populations. A score greater than 50% is considered an index of culturally sensitive materials, whereas a score less than 50% was considered an index of materials that are not culturally sensitive to the Arab or Jewish populations in Israel.

For the purpose of statistical comparison of CS between the materials written in Hebrew and those written in Arabic, each type of material underwent a separate chi-square test of independence. In addition, the chi-square test of independence was conducted to compare between the CS of the materials written in the 2 languages.

#### Qualitative Content Analysis

The second analysis method entailed qualitative content analysis [109,110], which focuses on the language characteristics and the content or contextual meaning of the text and provides knowledge and understanding of the phenomenon under study.

Qualitative content analysis relies on deciding what is going to be analyzed, forming the dataset to be collected, defining the unit or theme of analysis, classifying the content according to ideas that can be words or sentences, developing categories and creating subcategories, linking the interpretations with the basic theories of the study, defining categories with examples, drawing inferences on the basis of themes, and presenting the results [109,110].

In presenting the results related to each theme, we supported these ideas with typical secondary quotes that appeared frequently in most of the explanatory materials analyzed by the quantitative coding index.

In this study, qualitative content analysis is intended to explain and reinforce the researchers’ coding and to interpret the absence of indicators in the quantitative CS index.

Moreover, this method is designed to view the subjective interpretation of the content through a systematic classification process of identifying themes and categories that may affect or intensify the issue of CS.

The content analysis was based on comparison with the scientific literature on cervical cancer and the HPV vaccine. In addition, the materials were analyzed with reference to concepts from the field of health and risk communication: *CS* [12,15] and *transparency* in communicating texts about health issues [111].

### Reliability and Validity

This study is based on a variety of methods used to analyze rhetorical and aesthetic elements and scientific facts. The use of a combination of several research methods increases the reliability and validity of the findings [112-115] The CS index constructed by the researchers was based on Resnicow’s model of CS [12,15], a valid theoretical model. The researchers, among them experts in health communication and CS worked together to investigate the cultural attributes of the Arab Jewish populations in Israel in the context of the HPV vaccine, adapt these attributes to the index, define the dimensions, and formulate the questions. The researchers are able to read and analyze texts written in Hebrew and in Arabic. One of the researchers is a member of the Arab minority group, Arabic is her native language, and she can distinguish cultural uses of the language. The coders performed their coding separately, then compared their coding and found it to be almost identical. The third researcher reviewed all the findings after coding and gave his analysis of CS. Coding that was not equivalent was resolved through discussions between the research team members. A high level of interrater reliability (98%) emerged. An intercoder agreement coefficient exceeding 0.7 suggests an acceptable level of reliability [116].

Furthermore, Scott’s pi indicator was calculated by the reliability calculation for the masses, to reinforce the indicator of intercoder reliability. Scott’s pi value was 0.9685, thus ensuring valid internal coding reliability [117].

In the qualitative analysis of the texts, the researchers reviewed materials posted on the websites of the Ministry of Health and the Clalit HMO. Together, they determined the inclusion criteria and decided which materials would be included in the research. The major categories and themes emerging from the web-based explanatory materials were discussed and agreed upon [109,110].

## Results

### Cultural Sensitivity Index Implementation

This section describes the CS index implementation scores for the HPV vaccine materials published in Arabic and Hebrew on the websites of the Ministry of Health and the Clalit HMO. Implementation of the index included coding the nominal categorical indicators of each dimensions (surface and deep) with 1 of 2 values: yes/no (Multimedia Appendix 1) and calculating 2 scores: specific scores for the deep and surface dimensions and an overall CS index score (Multimedia Appendix 2).

### Surface Dimension–Specific Score

In most (78%) of the Arabic explanatory materials assessed, the *surface dimension* score was lower than 50%. Surface dimension score was higher than 50% for only 2 out of the 9 materials assessed. Conversely, surface dimension score was equal to or higher than 50% for all the materials (100%) written in Hebrew, starting from 73% up to 91% in some cases (33%).

The majority of the Arabic and Hebrew materials included some surface dimension indicators, such as mentioning the target audience (Arab population), use of suitable people (photos of Arab physicians recommending and explaining about the vaccine), language appropriate to the target audience (Arabic), compatibility with the literacy level (minimal use of scientific terminology), and presentation of the effectiveness and advantages of the recommended behavior (uptake of HPV vaccine).

Furthermore, most of the Arabic materials did not refer to the indicators of appropriate media channels (Arab-language television and radio stations or local websites targeting the Arab population), appropriate communicators (key Arab health and medical personnel), places suitable for transmitting the message (mosques, churches, and schools), and organizations acceptable to the minority target audience (educational originations and the Child and Family Care centers). In contrast, as mentioned, these indicators appeared in materials written in Hebrew, resulting in a surface dimension score equal to or higher than 70%. Moreover, most of the materials written in both languages, Hebrew and Arabic, did not address all the implications of vaccine uptake or the relevance of the vaccine or cervical cancer to both populations.

A chi-square test of independence of the surface dimensions reveals significant interaction between the explanatory materials written in Arabic and those written in Hebrew with respect to the following items: HPV vaccine, HPV vaccine for eighth-grade girls, vaccine to protect against cervical cancer caused by the papilloma virus, and a PowerPoint presentation about HPV vaccine.

### Deep Dimension–Specific Score

All the Arabic explanatory materials assessed scored below 50% on the *deep dimension* **.** Most of the materials did not refer to the social or normative values or to the environmental or cultural-ethical indicators.

Most of the materials that scored 20% on the deep dimension included 1 deep dimension indicator: either normative or social indicators. The normative indicator referred to Arab women’s preference for female gynecologists, particularly for Pap smear. The social indicator referred to providing explanations for giving the vaccine to males. These indicators were also referenced in some of the Hebrew informational materials.

Moreover, it is important to note that sexuality is related to the HPV vaccine even though the topics of sexuality and sexual discourse are known to be taboo among the Arab population. The data show that the Arabic explanatory materials made no reference whatsoever to the population’s moral concerns regarding sexual relations and promiscuity or to values and perceptions related to sexual relations before marriage.

In contrast, these indicators were referred to and mentioned in Hebrew explanatory materials, resulting in a deep dimension score exceeding 50% in most (78%) cases. Environmental factors, such as the impact of the sexual behavior of the nation’s majority population on the sexual concepts of the minority population, were absent from the informational materials in both languages.

A chi-square test of independence of the deep dimensions revealed significant interaction between the explanatory materials written in Arabic and those written in Hebrew with respect to the following items: information sheet before and after administering HPV vaccine, HPV vaccine, HPV vaccine for eighth-grade boys, and vaccine to protect against cervical cancer caused by the papilloma virus.

### Overall Cultural Sensitivity Index Score

The overall CS index score calculated for all the explanatory materials targeting the Arab minority points to a low level of CS, in contrast to the CS expressed in materials targeting the Jewish population. The low score reflects only a partial and superficial degree of CS and refers only to a minor portion of CS indicators. The overall CS index score based on the surface indicators, among them using Arabic, mentioning the Arab population, using Arab figures, and referring to the population’s low level of literacy, was higher than the score for the deep dimension indicators. The overall CS index score for deep dimension indicators included at most 1 deep indicator, without any reference to normative, social, cultural, traditional, and ethical indicators of the Arab minority population in Israel. Related issues affecting the decision-making process of this population regarding vaccines in general were not addressed, and reference to the issue of sexuality associated with the HPV vaccine was notably absent.

For the overall CS index score, a chi-square test of independence reveals significant interaction between the materials written in Arabic and those written in Hebrew on all the items except for HPV vaccine.

Moreover, a chi-square test of independence between CS and language (Hebrew vs Arabic) for all the explanatory materials revealed significant interaction on all the dimensions: deep dimensions, surface dimensions, and overall CS index score.

#### Qualitative Content Analysis of the Explanatory Materials on the Topic of the Human Papillomavirus Vaccine

The findings of the content analysis are presented in the form of a comparison between information given in the explanatory materials and information in the scientific literature. The findings reveal 2 main themes: (1) CS and the absence of any reference to the connection between sexuality and the HPV vaccine in the materials written in Arabic targeting the Arab minority, in contrast to those written in the Hebrew and (2) a lack of transparency in transmitting the information in both languages.

#### Cultural Sensitivity and the Absence of Reference to Connection Between Sexuality and Human Papillomavirus Vaccine in Explanatory Materials Targeting the Minority Population

The informational materials written in Arabic referred to only 2 indicators of deep CS: a normative indicator, referring to women’s preference for female gynecologists, particularly for Pap smear, and a social indicator explaining why a *female* vaccine is administered to males. The following main themes emerged: (1) women prefer female physicians to carry out tests, (2) the HPV vaccine prevents cancer among males, and (3) the HPV vaccine does not legitimize sexuality.

##### Women’s Preference for Female Physicians to Carry Out Tests

Women in the traditional conservative Arab society prefer not to be exposed to male doctors and would rather be examined by female physicians, especially in cases of infertility and gynecology that entail a variety of invasive tests [118]. The findings indicate that the explanatory informational materials referred to this normative issue by informing women they can ask for a female gynecologist to perform the Pap smear:

If you want to do the Pap smear, go to a female gynecologist in the clinic nearest to your home.

Clalit HMO, Arabic

##### The Human Papillomavirus Vaccine Prevents Cancer Among Males

The scientific literature indicates that some males are at high risk for developing various types of cancer because of infection by the papilloma virus, for example, anal cancer and other types of cancer more prevalent among men who have sex with men [119-121]. Men and women who are infected with HIV or other diseases marked by suppressed immune systems are also at increased risk of developing cancer related to the papilloma virus [122]. Yet, the explanatory materials did not specify a particular population of males at risk but rather explained why the vaccine is given to males in the context of protection against cancer:

The papilloma virus is liable to cause diseases (warts and cancerous growths) in men as well. Vaccinating men achieves two objectives: protecting them from diseases and protecting their sexual partners.

Clalit HMO, Arabic

The vaccine, now included in the basket of health services, is intended for women as well as for men and provides protection against cancer of the penis, anal cancer, vaginal cancer and throat cancer.

Clalit HMO, Arabic

Moreover, as mentioned above, the results show that the explanatory materials made no reference whatsoever to the connection between sexuality and the HPV vaccine. The Arabic explanatory materials described the vaccine against the papilloma virus as a vaccine that prevents cervical cancer and other types of cancer among males and females without any mention of the fact that the virus is transmitted only through sexual relations:

Why are boys vaccinated? For the same reason that girls are vaccinated: to protect girls and boys from cancerous diseases and from genital warts caused by the virus and to prevent passing the virus from one person to another

Ministry of Health, Hebrew, Arabic

##### The Human Papillomavirus Vaccine Does Not Legitimize Sexuality

The transmission of HPV by means of sexual contact aroused controversy regarding the vaccine. Many parents expressed concerns that the vaccine would raise the likelihood of sexual activity or would promote promiscuity [85-87]. The findings show that only the explanatory materials published in Hebrew refer to these concerns regarding sexuality among adolescents in the context of the vaccine. Most of the current materials explain that there is no relationship between the vaccine and adolescent sexual activity and that the vaccine is intended to promote sexual health among adolescents before they become sexually active:

The risk of uncontrolled sexual activity among young girls who receive the HPV vaccine is no greater than among young girls or women who do not receive the vaccine.

Clalit HMO, Hebrew

Research studies show that the vaccine does not encourage earlier commencement of sexual activity.

Ministry of Health, Hebrew

#### Cultural Sensitivity and Lack of Transparency Regarding the Controversy Surrounding the Human Papillomavirus Vaccine

The findings show that the explanatory informational materials written in both languages, Hebrew and Arabic, state that the HPV vaccine is totally safe and effective but make no reference to a variety of other factors affecting safety and effectiveness. Moreover, the findings show that the explanatory informational materials attempt to depict cervical cancer as a serious disease that endangers public health, whereas the HPV vaccine is portrayed as the ultimate solution for cervical cancer without any suggested alternatives.

The following main themes emerged: (1) total effectiveness of the HPV vaccine, (2) absolute safety and minor side effects of the HPV vaccine, (3) the HPV vaccine defeats cervical cancer, (4) there is no suitable alternative for the vaccine, (5) cervical cancer is quite prevalent, and (6) the papilloma virus definitely causes cervical cancer.

##### Total Effectiveness of the Human Papillomavirus Vaccine

Numerous clinical trials have proven the effectiveness of the HPV vaccine (87%-100%) [34,35,123]. Nevertheless, the literature still contains studies pointing to a variety of factors that can affect vaccine effectiveness, such as age, type of virus strain, and the individual’s immune system [40-44]. Analysis of the explanatory materials from the Ministry of Health and Clalit HMO websites indicates that none refers to these factors and all report that the HPV vaccine is “extraordinarily effective”:

The Gardasil vaccine is 100% effective in preventing cervical cancer and in preventing genital warts caused by the papilloma virus. In addition, the vaccine is 99% effective in preventing precancerous lesions caused by four strains of the papilloma virus (6, 11, 16 and 18).

Clalit HMO, Hebrew

Evidence from 20 research studies in nine countries: The HPV vaccine provides 100% protection from infection by the strains covered by the vaccine. The vaccine reduced the rate of virus infection among girls aged 14-19 by 64%. The vaccine reduced the rate of genital warts among boys aged 14-19 by 31%.

Ministry of Health, Hebrew/Arabic

##### Absolute Safety and Minor Side Effects of the Human Papillomavirus Vaccine

Numerous studies have pointed to a spectrum of side effects of the HPV vaccine, ranging from common mild vaccine effects such as pain at the injection site [46] to more severe effects such as chronic pain syndrome and chronic fatigue [59]; Guillain-Barré syndrome, spinal cord inflammation, and venous blood clots [55]; autoimmune diseases such as ovarian failure [56-58]; and postural orthostatic tachycardia syndrome with chronic pain [59].

The web-based explanatory materials noted mild side effects that are not critical or severe and that pass within a short period of time as well as a limited number of more serious side effects that were described as rare. The following quotations describe the vaccine’s mild side effects:

The vaccine can be accompanied by side effects that pass quickly… redness, pain and swelling at the injection site… fever, general sick feeling, muscle and joint pains, and digestive disturbances.

Clalit HMO, Hebrew

Local side effects are redness, pain and swelling at the injection site. General effects include fainting, dizziness, nausea, headaches, fever. Diarrhea and vomiting are also possible.

Ministry of Health, Hebrew/Arabic

In the following quotations, more serious side effects are described as rare. For example, *anaphylaxis* is described as follows:

Infrequently, a rapid and severe allergic reaction known as anaphylaxis is liable to occur. This reaction is characterized by shortness of breath, overall itching or rash, drop in blood pressure, rapid pulse, dizziness, abdominal pain, or vomiting and diarrhea… This reaction is rare and comes on quickly, which is the reason for the 15-minute wait in the clinic after the injection. If necessary, the clinic is equipped with an effective treatment kit for emergency treatment.

Clalit HMO, Hebrew

Moreover, *complex regional pain syndrome* and *chronic fatigue syndrome* are depicted as effects that are unrelated to the HPV vaccine:

No causal relation has been found between the HPV vaccine and these syndromes. The rate of these syndromes is 150 in every 1,000,000 girls aged 10-19 and was no higher among girls and women who received the vaccine than among those that did not.

Ministry of Health, Hebrew/Arabic

##### The Human Papillomavirus Vaccine Defeats Cervical Cancer

There is research evidence for skepticism regarding the long-term preventive effectiveness of the HPV vaccine [124]. Yet despite this, all the explanatory materials portrayed the vaccine as an effective defense that defeats the silent illness and helps prevent it. For example:

The papilloma vaccine: the effective and safe way to prevent cancer.

Ministry of Health, Hebrew/Arabic

The HPV vaccine protects against cervical cancer and also against genital warts.

Ministry of Health, Hebrew/Arabic

##### There Is No Suitable Alternative for the Vaccine

Many research studies pointed to ambiguous and vague reports regarding alternatives for the HPV vaccine and raised concerns regarding the effectiveness of these alternatives in preventing cervical cancer, including Pap smears [94] and condoms [125]. The findings of this study also indicate that most of the explanatory materials portray the HPV vaccine as the “exclusive preventive measure” against cervical cancer caused by the virus, with no reference to any alternatives. For example, the materials raise doubts about the effectiveness of condom use:

Condoms do not prevent infection with this disease. Sometimes the virus is found in warts on the genitals, so that infection can be caused by direct contact with the skin containing warts or Condyloma.

Clalit HMO, Hebrew

Using condoms during sexual relations can help prevent infection with the papilloma virus. But because condoms do not cover the entire genital area and are usually put on after sexual contact has begun, they do not constitute a guarantee against HPV infection.

Ministry of Health, Hebrew/Arabic

In addition, despite the need for ongoing Pap smear after receiving the vaccine [25,126,127] and despite the typical effectiveness of the Pap smear in reducing cervical cancer morbidity worldwide [128,129], the explanatory materials played down this information as follows:

Pap smear reveal precancerous cervical lesions, not to prevent the disease.

Clalit HMO, Hebrew

##### Cervical Cancer Is Quite Prevalent

The incidence and mortality rates for cervical cancer vary significantly in different countries worldwide, with the highest rates of incidence and mortality specific to third-world countries [130]. The findings show that the explanatory materials describe the morbidity and mortality rates in vague terms and not specific to any particular country or region. Thus, the high incidence rates shown are more relevant to developing nations and not to developed countries such as Israel [130,131] or other Western countries where cervical cancer is practically nonexistent [132]. It is important to emphasize that the prevalence of cervical cancer in Israel is lower than that in other Western countries [133,134], a fact that most of the explanatory materials do not mention. Indeed, the materials describe cervical cancer as a “common threat,” as in the following quotations:

Cervical cancer kills 290,000 women worldwide.

Clalit HMO, Arabic

According to the World Health Organization, around 300 million people worldwide are infected with HPV each year, 490,000 women contract cervical cancer and around 230,000 die from cervical cancer each year.

Clalit HMO, Hebrew

The explanatory materials also provide specific figures for cervical cancer in Israel. Yet, most provide morbidity rates that have not been updated for many years:

Between 1990 and 2004, 150 new cases of cervical cancer were diagnosed in Israel each year. Sixty women in Israel die of the disease each year.

Clalit HMO, Hebrew

Around 180 women are diagnosed each year in Israel. Every year around 80 women die of cervical cancer.

Ministry of Health, Hebrew/Arabic

##### The Papilloma Virus Definitely Causes Cervical Cancer

The scientific literature indicates that infection with the papilloma virus does not indubitably cause cancer of the cervix. Studies have shown that some papilloma virus infections go away on their own within a period of a year or more without any long-term consequences. Chronic HPV infections raise the chances of developing cervical cancer, though only a small portion of these chronic infections ultimately develop into cancer [135-137]. The study’s findings show that most of the explanatory materials of all the health authorities depicted infection with the papilloma virus as the main and primary factor that promotes the immediate and certain development of cervical cancer. For example:

99.7% of all cases of cervical cancer are related to HPV… The papilloma virus is responsible for 90% of cases of anal cancer in men and women, 70% of cases of cancers of the mouth and throat, 70% of vaginal and genital cancers, and 50% of cases of cancer of the penis.

Ministry of Health, Hebrew/Arabic

## Discussion

The research findings related to the web-based explanatory materials that were designed to promote the use of HPV vaccine as well as those that appealed to 2 population groups: Arabs and Jews. The analysis of CS in the explanatory materials is based on the definitions of the surface and deep dimensions proposed by Resnicow et al [12,15].

A comparison between the explanatory informational materials targeting the Jewish population and those targeting the Arab population reveals differences in the expression of CS dimensions. With respect to surface dimension, the results of the study reveal more CS in the explanatory materials targeting the Jewish population. Informational materials written in Hebrew are more culturally sensitive to the target population based on the following indicators: (1) appropriate media channel—the websites of the examined health authorities are considered an appropriate media channel for the Jewish population because the Jewish population has greater access to the internet than the Arab population [138,139], (2) place and organizations suitable for transmitting messages—the Ministry of Health and the Clalit HMO are regarded as suitable because they deliver information in the target audience’s language [140], and (3) reliable suitable communicators—the Jewish population places more trust in the Ministry of Health than the Arab population [141]. In contrast, the findings show that the explanatory materials written in Arabic demonstrated only partial CS to the Arab minority, mainly with respect to the use of persons and language appropriate for this population. These findings are in line with those of other studies that examined CS in materials intended to influence other health behaviors among other minority populations in Western countries [142,143].

In addition, the informational materials written in the different languages treated the literacy level in their target audiences differently. Resnicow contends that culturally adapting materials to a specific population group requires taking into consideration that group’s ability to search for, read, and understand information [12,15]. The level of health literacy is known to be related to many factors, among them education, socioeconomic status, marital status, and others [144,145]. Nevertheless, every population group, even those where the literacy level is not high, can make a decision if the informational materials are transmitted in a transparent and culturally sensitive manner [15]. This study found that the lack of consideration of the literacy level of the Arab population (which is lower than that of the Jewish population [146] can block the population from obtaining, understanding, and using information, thus having a negative impact on the decision-making process regarding HPV vaccination.

With respect to the deep dimension, a limited portion of the explanatory materials written in Arabic made reference to the deep dimensions of the cultural attributes of the Arab population, such as the preference for female gynecologists in performing Pap smears [118] and the explanations for giving males a vaccine intended to prevent cervical cancer [147,148].

One interesting finding is that all the materials written in Hebrew refer to the sexual context associated with the HPV vaccine, representing high deep CS. Nevertheless, the lack of references in the materials targeting the Arab population to the socially and culturally sensitive topic of the connection between engaging in sexual relations and giving the HPV vaccine is paradoxical. The topic of sexuality is seemingly considered taboo in conservative Arab society [100]. Arab society worldwide [99,101] and in Israel [149] does not consider it legitimate either for men or for women to engage in sexual relations before marriage. In patriarchal societies, it is not acceptable for women to have sexual aspirations or desires before marriage [150,151]. Hence, the designers of the explanatory materials targeting women positioned the vaccine in the context of cancer, without mentioning sexual relations [152,153]. Similarly, the appeal to men in these materials was made in the context of protection against various forms of cancer, without reference to any sexual context. It is reasonable to assume that the designers of the explanatory materials chose to disregard the sexual context to avoid undermining the taboo and to adapt the materials to the cultural and religious norms of the target population [154]. Hence, it can be argued that the explanatory materials demonstrated sensitivity to the culture of the target population.

Yet, conversely, based on analysis of the deep dimensions of the explanatory materials, it is possible to claim that this sensitivity is only **s**uperficial and that, in practice, no reference is made to the cultural attributes of the target population. According to the deep dimensions as defined in the model developed by Resnicow et al [12,15], such materials should refer to ethical considerations and to the concerns of the population regarding carrying out behavior that is not in keeping with their cultural and normative customs. On the basis of this definition, the explanatory materials designed for the Arab population should have referred to the ethical concerns of this conservative population regarding the vaccine and its implications with respect to encouraging sexual relations and sexual promiscuity before marriage [102,155,156]. As noted above, this was missing from the materials in practice. This may accentuate the lack of transparency in the transmission of full information about the deep dimension indicators.

This unique finding of this study can explain the high rate of HPV vaccination compliance among the Arab minority population, a rate that is more than double the rate among the Jewish population [157]. One reason for this discrepancy may be related to the lack of CS in the informational materials. Lack of information about the relationship between sexuality and vaccination and the absence of references to moral concerns about sexual behavior following vaccination result in an uninformed and unwise decision-making process. Hence, the Arab population remains unaware that the HPV virus is transmitted through sexual relations, a fact that is liable to lower their compliance rate [72,73,75,79].

Moreover, the textual analysis in this study indicates that the texts are not fully transparent regarding the surface dimensions of the CS index in the context of the vaccine’s effectiveness and safety or the relevance of cervical cancer to the target population. Providing transparent health information regarding the HPV vaccine, including a comprehensive description of the scientific controversy surrounding the vaccine’s safety and effectiveness [50,127,158,159] and figures showing the low prevalence of the disease in Israel relative to the rest of the world [131,133], would enable each individual to make an independent and intelligent decision based on informed consent. Furthermore, transparent health information about cervical cancer, the relevance of this illness to the population, and the vaccine’s effectiveness is likely to reduce individuals’ perceived risk of developing cervical cancer and decrease their intentions to obtain the HPV vaccine [160].

This finding may be another reason for the high compliance rate among the Arab population that is related to the lack of transparency regarding the scientific controversy surrounding the vaccine. The fact that the Jewish population is more exposed to the scientific controversy surrounding the vaccine’s side effects through social networking discussions and searching scientific articles [161] can explain the compliance gap between the 2 population groups.

The current literature on risk communication clearly shows that when the media and the health authorities do not provide complete and transparent information in response to the public’s fears and moral concerns, this can have a boomerang effect [33,162], leading the public to doubt the information’s reliability or to suspect that the authorities are hiding information. Moreover, in the age of new media, the public is active and exposed to a great deal of information, underscoring the importance of CS, transparency, and providing full information. If the public does not receive what it seeks from the official authorities, it will turn to other sources of information.

Therefore, the criterion of transparency is not only a criterion for *high level health communication* but also a criterion used in this paper to reveal the lack of full CS in promoting the HPV vaccine among the conservative Arab population. Hence, to achieve full CS, it is important to treat the target audience with respect and to provide them with all the information relevant to the vaccine to enable them to make culturally appropriate decisions [163,164].

### Limitations

This study examined web-based explanatory materials published on the websites of official health authorities in Israel. The research did not include flyers, pamphlets, posters, or information brochures that might be distributed in health clinics or other health institutions. Nevertheless, today, the HPV vaccine is not given in clinics or other health institutions because it is part of the routine school vaccine program. Therefore, it is reasonable to assume that the study included most of the existing informational materials.

As stated, the study included only the informational materials appearing on websites. Considering the 70% internet access rate of the Israeli population in general and the 50% access rate of the Arab population in particular, it is possible that not all of the population was exposed to these materials. However, it is reasonable to assume that if the public needs information about the HPV vaccine or any other health information, it prioritizes the official health authorities’ websites.

Moreover, this study was based mainly on qualitative and quantitative content analysis of explanatory materials targeting different population groups in Israel. The research did not consider how these materials were received and accepted by the public, that is, it did not assess their effectiveness among the public. Future research on audience analysis is recommended to examine how the public receives the explanatory materials. In addition, for the deep dimensions, the number of indicators was quite small (5 indicators). Therefore, drawing statistical conclusions was problematic in the comparison between materials written in Hebrew and those written in Arabic.

The research focused on official health authorities’ informational materials and did not generalize to the materials of other countries. Other studies might be comparative and study the transfer of information between different countries.

### Conclusions

The public health authorities have 2 main objectives in the context of vaccinations. One is to raise the vaccination rates and the other is to provide full and culturally sensitive information to give the public the tools to make intelligent decisions. The findings of this study indicated that despite the high uptake rate for HPV vaccination in the Arab population, the health authorities did not exercise full transparency and CS in transmitting the association between engaging in sexual relations and necessity of the vaccination. Thus, the major challenge for the health authorities is to find ways to implement the objective of communicating information about the vaccination in a way that is transparent and culturally sensitive, even if this raises questions and fears among the public deriving from their culture.

Specifically, explanatory health materials in general and those promoting the HPV vaccine in particular must provide all the relevant information available in the literature today in a manner that is comprehensive, detailed, culturally sensitive, and based on scientific evidence to enable each individual to make an independent and intelligent decision based on informed consent.

The main recommendation of this study is to make CS the first priority in designing explanatory materials targeting minority population groups. Referring to the cultural attributes of ethnic minority groups in such explanatory materials is important for empowering the population and stimulating open and appropriate discourse.

In addition, because the media have the power to influence a person’s health views and behaviors, the research recommends using media strategies that implement transparency in providing complete information.

## Appendix

Multimedia Appendix 1 Coding of categorical indicators of surface dimension and coding of categorical indicators of deep dimension.

Multimedia Appendix 2 Human papillomavirus vaccine materials published in Arabic and Hebrew: details and index scores.

## Abbreviations

CS: cultural sensitivity
HMO: health maintenance organization
HPV: human papillomavirus
Pap: Papanicolaou
WHO: World Health Organization
