# Supplementary material for: Case of Paradoxical Cultural Sensitivity: Mixed Method Study of Web-Based Health Informational Materials About the Human Papillomavirus Vaccine in Israel
Source: J Med Internet Res. 2019 May 17;21(5):e13373. doi: 10.2196/13373 (PMC6543802; doi:10.2196/13373)
Supplement: Multimedia Appendix 1 [file jmir_v21i5e13373_app1.docx]

Table 2a: Coding of categorical indicators of surface dimension (First answer refers to Arabic documents/second answer refers to Hebrew documents).

| **HPV vaccine materials**  **Surface Dimension Indicators** | **Cervical Cancer (first document)** | **Cervical Cancer (second document)** | **Human Papilloma Virus Vaccine** | **Information Sheet Before and After Administering HPV Vaccine** | **Human Papilloma Virus Vaccine** | **HPV Vaccine for Eighth Grade Girls** | **HPV Vaccine for Eighth Grade Boys** | **Vaccine to Protect Against Cervical Cancer Caused by Papilloma Virus** | **PowerPoint Presentation about HPV Vaccine** |
| --- | --- | --- | --- | --- | --- | --- | --- | --- | --- |
| Is the target population mentioned in the title or the content, either directly or indirectly? | **Yes/Yes** | **Yes/Yes** | **Yes/Yes** | **Yes/Yes** | **Yes/Yes** | **Yes/Yes** | **Yes/Yes** | **Yes/Yes** | **Yes/Yes** |
| Do the materials use people who are appropriate for, familiar to and acceptable to the target population? | **Yes/No** | **Yes/No** | **Yes/No** | **Yes/Yes** | **Yes/Yes** | **No/Yes** | **Yes/No** | **No/No** | **No/No** |
| Do the materials use the mother tongue of the target population? | **Yes/Yes** | **Yes/Yes** | **Yes/Yes** | **Yes/Yes** | **Yes/Yes** | **Yes/Yes** | **Yes/Yes** | **Yes/Yes** | **Yes/Yes** |
| Are the materials formulated in a manner appropriate to the literacy level of the target population? | **Yes/Yes** | **Yes/Yes** | **Yes/Yes** | **Yes/Yes** | **No/Yes** | **Yes/Yes** | **No /yes** | **Yes/Yes** | **No/ Yes** |
| Do the materials explain the relevance of cervical cancer to the target population? | **No/yes** | **Yes/Yes** | **No/Yes** | **No/No** | **No/No** | **No/No** | **No/No** | **No/Yes** | **No/No** |
| Do the materials explain the effectiveness of the HPV vaccine against cervical cancer in the target population? | **Yes/Yes** | **Yes/Yes** | **Yes/Yes** | **Yes/Yes** | **Yes/Yes** | **Yes/Yes** | **Yes/Yes** | **Yes/Yes** | **Yes/Yes** |
| Do the materials explain the implications of the HPV vaccine against cervical cancer in the target population (side effects, complications, etc.)? | **Yes/Yes** | **No/yes** | **No/No** | **No/No** | **Yes/Yes** | **No/No** | **No/No** | **No/No** | **No/No** |
| Are appropriate media channels used that are familiar to the target population? | **No/Yes** | **No/Yes** | **No/Yes** | **No/Yes** | **No/Yes** | **No/Yes** | **No/Yes** | **No/Yes** | **No/Yes** |
| Are the materials distributed in places most suitable for the target population (churches, schools, medical clinics versus websites)? | **No/Yes** | **No/Yes** | **No/Yes** | **No/Yes** | **No/Yes** | **No/Yes** | **No/Yes** | **No/Yes** | **No/Yes** |
| Do the materials use a reliable communicator who is appropriate for the target population? | **No/Yes** | **No/Yes** | **No/Yes** | **No/Yes** | **No/Yes** | **No/Yes** | **No/Yes** | **No/Yes** | **No/Yes** |
| Was the organization that transmitted the information from the same ethnic group (Arab/Jewish nationality)? | **No/Yes** | **No/Yes** | **No/Yes** | **No/Yes** | **No/Yes** | **No/Yes** | **No/Yes** | **No/Yes** | **No/Yes** |

Table 2b: Coding of categorical indicators of deep dimension (First answer refers to Arabic documents/second answer refers to Hebrew documents).

| **HPV vaccine materials**  **Deep Dimension Indicators** | **Cervical Cancer (first document)** | **Cervical Cancer (second document)** | **Human Papilloma Virus Vaccine** | **Information Sheet Before and After Administering HPV Vaccine** | **Human Papilloma Virus Vaccine** | **HPV Vaccine for Eighth Grade Girls** | **HPV Vaccine for Eighth Grade Boys** | **Vaccine to Protect Against Cervical Cancer Caused by Papilloma Virus** | **PowerPoint Presentation about HPV Vaccine** |
| --- | --- | --- | --- | --- | --- | --- | --- | --- | --- |
| Do the materials refer to the moral concerns of the target population regarding the HPV vaccine (increased sexual relations, encouraging promiscuity)? | **No/Yes** | **No/Yes** | **No/Yes** | **No/Yes** | **No/Yes** | **No/Yes** | **No/Yes** | **No/Yes** | **No/Yes** |
| Do the materials refer to the cultural concepts regarding cervical cancer and the HPV vaccine (preference for women practitioners for PAP smears or other gynecological exams)? | **Yes/No** | **No/No** | **No/No** | **No/No** | **No/No** | **No/No** | **No/No** | **No/No** | **Yes/No** |
| Do the materials refer to social concepts regarding cervical cancer and the HPV vaccine (cervical cancer is a female disease, reasons for giving the vaccine to men)? | **No/Yes** | **Yes/Yes** | **Yes/No** | **No/Yes** | **No/Yes** | **No/No** | **No/Yes** | **No/Yes** | **No/Yes** |
| Do the materials refer to environmental concepts (influence of the Arab/Jewish population, socioeconomic status)? | **No/No** | **No/No** | **No/No** | **No/No** | **No/No** | **No/No** | **No/No** | **No/No** | **No/No** |
| Do the materials refer to value concepts (religion, fatalism, sexual relations before marriage)? | **No/Yes** | **No/Yes** | **No/Yes** | **No/Yes** | **No/Yes** | **No/Yes** | **No/Yes** | **No/Yes** | **No/Yes** |
