# Supplementary material for: Case of Paradoxical Cultural Sensitivity: Mixed Method Study of Web-Based Health Informational Materials About the Human Papillomavirus Vaccine in Israel
Source: J Med Internet Res. 2019 May 17;21(5):e13373. doi: 10.2196/13373 (PMC6543802; doi:10.2196/13373)
Supplement: Multimedia Appendix 2 [file jmir_v21i5e13373_app2.docx]

Table 3: HPV Vaccine Materials Published in Arabic and Hebrew: Details and Index Scores.

| **Material Title** | **Year Updated** | **Health Authority** | **SDS - Surface Dimension Score (11 items)** | | | **DDS - Deep Dimension Score (5 items)** | | | **Overall CS Index Score ((SDS+DDS)/2)** | | |
| --- | --- | --- | --- | --- | --- | --- | --- | --- | --- | --- | --- |
|  |  |  | **Arabic**  **(n)** | **Hebrew**  **(n)** | $\boldsymbol{\chi}_{\boldsymbol{(1)}}^{\boldsymbol{2}}$  **(*p*)** | **Arabic**  **(n)** | **Hebrew**  **(n)** | $\boldsymbol{\chi}_{\boldsymbol{(1)}}^{\boldsymbol{2}}$  **(*p*)** | **Arabic** | **Hebrew** | $\boldsymbol{\chi}_{\boldsymbol{(1)}}^{\boldsymbol{2}}$  **(*p*)** |
| Cervical Cancer (first document) | 2013 | Clalit HMO | 55% (6) | 91% (10) | 3.67  (.06) | 20% (1) | 60% (3) | 1.67 (0.20) | 37% | 75% | 4.80 (0.03)* |
| Cervical Cancer (second document) | 2014 | Clalit HMO | 55% (6) | 91% (10) | 3.67  (.06) | 20% (1) | 60% (3) | 1.67 (0.20) | 37% | 75% | 4.80 (0.03)* |
| Human Papilloma Virus Vaccine | 2013 | Ministry of Health | 45% (5) | 82% (9) | 3.14  (0.08) | 20% (1) | 40% (2) | 0.48 (0.49) | 33% | 61% | 3.14 (0.08) |
| Information sheet before and after administering HPV vaccine | 2013 | Ministry of Health | 45% (5) | 82% (9) | 3.14  (0.08) | 0% (0) | 60% (3) | 4.29 (0.04)* | 23% | 71% | 6.15 (0.01)* |
| Human Papilloma Virus Vaccine | 2013 | Ministry of Health | 45% (5) | 91% (10) | 5.24 (0.02)* | 0% (0) | 60% (3) | 4.29 (0.04)* | 23% | 75% | 8.13 (0.00)* |
| HPV vaccine for eighth grade girls | 2013 | Ministry of Health | 36% (4) | 82% (9) | 4.70  (0.03)* | 0% (0) | 40% (2) | 2.50 (0.12) | 18% | 61% | 6.15 (0.01)* |
| HPV vaccine for eighth grade boys | 2015 | Ministry of Health | 36% (4) | 73% (8) | 2.93  (0.09) | 0% (0) | 60% (3) | 4.29 (0.04)* | 18% | 66% | 6.15 (0.01)* |
| Vaccine to protect against cervical cancer caused by the papilloma virus | 2014 | Ministry of Health | 36% (4) | 82% (9) | 4.70  (0.03)* | 0% (0) | 60% (3) | 4.29 (0.04)* | 18% | 71% | 8.00 (0.01)* |
| PowerPoint presentation about HPV vaccine | 2015 | Ministry of Health | 27% (3) | 73% (8) | 4.55  (0.03)* | 20% (1) | 60% (3) | 1.67 (0.20) | 24% | 66% | 6.15 (0.01)* |
| **Total** | | | SDS | | 31.06 (<0.001)** | DDS | | 21.00 (<0.001)** | Overall CS Index Score | | 47.34  (<0.001)** |
| * *P*<0.05 ** *P*<0.001 | | | | | | | | | | | |
